# World Endoscopy Organization Position Statements for Artificial Intelligence in Endoscopic Diagnosis of Gastric Epithelial Neoplasia

**DOI:** 10.1111/den.70144

**Published:** 2026-04-08

**Authors:** Mitsuhiro Fujishiro, Naomi Kakushima, Seiichiro Abe, Hon Chi Yip, Xuemei Liu, Gwan Ha Kim, Mathieu Pioche, George Adel Cortas, Kazuki Sumiyama, Vitor Nunes Arantes, Fabian Emura, Mário Dinis‐Ribeiro

**Affiliations:** ^1^ Department of Gastroenterology in Organ Pathophysiology Program, Graduate School of Medicine The University of Tokyo Tokyo Japan; ^2^ Division of Gastroenterology, Department of Medicine Showa Medical University School of Medicine Tokyo Japan; ^3^ Endoscopy Division National Cancer Center Hospital Tokyo Japan; ^4^ Division of Upper GI and Metabolic Surgery, Department of Surgery, Faculty of Medicine The Chinese University of Hong Kong Hong Kong China; ^5^ Department of Gastroenterology, Digestive Disease Hospital Affiliated Hospital of Zunyi Medical University Zunyi China; ^6^ Department of Internal Medicine, Pusan National University College of Medicine and Biomedical Research Institute Pusan National University Hospital Busan Korea; ^7^ Hepatogastroenterology Division Edouard Herriot Hospital, Hospices Civils de Lyon Lyon France; ^8^ Saint George Hospital University Medical Center, Faculty of Medicine, University of Balamand Beirut Beirut Lebanon; ^9^ Department of Endoscopy, School of Medicine The Jikei University Tokyo Japan; ^10^ Endoscopy Unit, Alfa Institute of Gastroenterology School of Medicine, Federal University of Minas Gerais, Hospital Mater Dei Contorno Belo Horizonte Brazil; ^11^ Digestive Health and Liver Diseases, Miller School of Medicine University of Miami Coral Gables Florida USA; ^12^ Department of Gastroenterology Portuguese Institute of Oncology of Porto Porto Portugal

**Keywords:** artificial intelligence, early gastric cancer, endoscopic diagnosis, World Endoscopy Organization

## Abstract

The World Endoscopy Organization (WEO) has prepared position statements for artificial intelligence (AI) in endoscopic diagnosis of gastric epithelial neoplasia as part of activities in the Stomach and Duodenal Diseases Committee. Gastric cancer is still a major cause of cancer death globally, and endoscopy plays a crucial role for early detection and early treatment to improve patients' quality of life as well as to save patients' lives. Artificial intelligence (AI) is an emerging technology to have the potential to increase endoscopic diagnostic yields dramatically, but evidence in clinical use is still insufficient, only from advanced institutions in certain countries. Thus, we developed three, three, and four position statements regarding computer‐aided detection, computer‐aided diagnosis, and promotion of research, respectively, for better understanding of the present standpoints and future perspectives of AI in endoscopic diagnosis of gastric epithelial neoplasia. AI in the stomach must be helpful to ensure the quality of endoscopy and to increase diagnostic accuracy, but it is still controversial in terms of cost‐effectiveness. In addition, it is necessary to develop AI for endoscopic diagnosis of not only gastric epithelial neoplasia but also all kinds of neoplastic lesions and the other alert lesions in the upper gastrointestinal tract in order to apply AI in the entire procedure of esophagogastroduodenoscopy (EGD). Furthermore, developing AI for risk stratification to know the best timing of EGD surveillance is warranted as future agenda.

**Abbreviations**
AI: artificial intelligence
AUC: area under the curve
BERN: Barrett's esophagus‐related neoplasia
CADe: computer‐aided detection
CADx: computer‐aided diagnosis
CI: confidence interval
DL: deep learning
EGC: early gastric cancer
EGD: esophagogastroduodenoscopy
ESCC: esophageal squamous cell carcinoma
ESCN: Esophageal squamous cell neoplasia
ESD: endoscopic submucosal dissection
GA: gastric atrophy
GEN: gastric epithelial neoplasia
GI: gastrointestinal
Hp:
*Helicobacter pylori*

ICER: incremental cost‐effectiveness ratio
IM: intestinal metaplasia
IQCS: intelligent quality‐control system
LCI: linked color imaging
NBI‐ME: magnifying endoscopy with narrow band imaging
MSI: microsatellite instability
OLGA: operating link for gastritis assessment
OLGIM: operating link for gastric intestinal metaplasia
RCT: randomized controlled trial
WEO: World Endoscopy Organization
WLI: white light imaging

## 1 Introduction

The clinical use of artificial intelligence (AI) in gastroenterology has rapidly expanded as a novel tool to support practitioners in detecting and characterizing gastrointestinal (GI) abnormalities. Early AI development in gastroenterology focused primarily on colorectal polyp and tumor detection, and AI systems for colonoscopy continue to advance, supported by a growing body of scientific evidence and clinical trials, as summarized in recent World Endoscopy Organization (WEO) position statements [1].

The application of AI in early gastric cancer (EGC) during esophagogastroduodenoscopy (EGD) is still limited to highly specialized centers, and in most countries and regions, AI remains investigational with uncertain benefits, risks, and burden. Even so, AI is expected to become an auxiliary tool in routine EGD in the near future, with the potential to improve detection accuracy and reduce diagnostic errors [2]. To assess the status of AI in the endoscopic screening and characterization of gastric epithelial neoplasia (GEN), we developed WEO position statements aiming to guide practitioners to understand whether to incorporate these innovative tools into routine endoscopy practice because gastric cancer is one of the most common cancers worldwide with more than 1,089,000 new cases and 769,000 patients died of gastric cancer worldwide in 2020 [3].

## 2 Procedures for the Preparation of the Position Statements

The first face‐to‐face meeting of the WEO Stomach and Duodenal Diseases Committee 2024–2026, chaired by MF, was held at ENDO 2024 in Seoul, Korea (July 2024). At this meeting, the committee agreed to develop WEO position statements on AI diagnosis of GEN, given the limited evidence but high potential of AI to transform endoscopic practice and the substantial global burden of gastric cancer, particularly in Asia. A second in‐person meeting took place at UEGW 2024 in Vienna (October 2024), where the committee confirmed the structure of the paper, comprising three sections: computer‐aided detection (CADe) (led by NK), computer‐aided diagnosis (CADx) (led by SA), and Promotion of Research (led by HCY and XL). Drafts of each section, including statements, explanations, and references based on literature searches, were circulated and revised via email among the developing committee members (all the listed authors, 1 from North America, 2 from Europe, 7 from Asia, 1 from Latin America, and 1 from the Middle East) by DDW 2025 in San Diego (May 2025). The drafts were then further discussed during the third face‐to‐face meeting at DDW 2025 to finalize the statements and explanations for the first voting [4]. Voting among 18 WEO Stomach and Duodenal Committee members in total was conducted anonymously by using the SurveyMonkey platform by the end of May 2025. The members were asked to indicate their thoughts for each statement by using a Likert scale with five possible answers (strongly agree, agree, neither agree nor disagree, disagree, strongly disagree). They were also asked to make comments in a free‐text box per each statement, if needed. Consensus was confirmed when 80% or more chose either “strongly agree” or “agree.” Statements that did not reach the consensus threshold during the voting processes were either removed or modified in accordance with discussion after each voting process. We planned more than one round voting if the consensus was not obtained in some statements. As the results of first round voting for 10 statements (3 CADe statements, 3 CADx statements, and 4 Promotion of research statements), all of them survived without discard as shown below.

## 3 Position Statements With Explanations

### 3.1 Computer‐Aided Detection

Statement 1.1 We recommend developing an AI system for ensuring the quality of EGD to reduce blind spots and increase the detection of GEN. (Strongly agree 61.1%, Agree 38.9%).

Statement 1.2 CADe for GEN is likely to improve EGD effectiveness by reducing GEN miss rates, thereby increasing GEN detection. (Strongly agree 38.9%, Agree 55.6%, Neither agree nor disagree 5.6%).

Statement 1.3 Health‐care delivery systems and authorities should evaluate the cost‐effectiveness of CADe for GEN to support its use in clinical practice. (Strongly agree 77.8%, Agree 22.2%).

To minimize missed lesions and improve GEN detection during EGD, systematic observation of the entire stomach is essential. However, full visualization is often difficult due to the stomach's complex anatomy, including folds, curvatures, and angulated regions. These blind spots, particularly in areas such as the fundus and antrum, can obscure small or flat early‐stage GEN, increasing the risk of oversight. Indeed, 5%–26% of gastric lesions are reported to be missed during EGD [5, 6]. The ability of AI to systematically highlight and ensure the coverage of all gastric regions helps mitigate this limitation, enhancing the accuracy of GEN detection [7]. Endoscopist factors such as quick inspection, operator distraction, and anatomically challenging areas prone to endoscopic blind spots can affect the effectiveness of EGD. Among these factors, thorough avoidance of blind spots is essential for ensuring optimal detection of GEN [8].

The WISENSE system, a real‐time convolutional neural network, was able to detect blind spots. In a randomized controlled trial (RCT) involving 324 patients, the blind‐spot rate was significantly lower in the WISENSE group than in the control group (5.9% vs. 22.5%, *p* < 0.001) [9]. An AI system known as ENDOANGEL was developed to integrate real‐time blind‐spot monitoring with EGC detection. In a multicenter trial involving 1050 patients, the AI‐assisted group exhibited significantly fewer blind spots compared with the control group (mean 5.38 vs. 9.82, *p* < 0.001) [10]. In a recent, single‐center, tandem RCT comparing the updated model of this system (ENDOANGEL‐LD) in 1014 patients, the miss rate of GEN, especially low‐grade dysplasia, was significantly lower in the AI‐first group than in the non‐AI‐first group (6.1%, 95% confidence interval [CI] 1.6%–17.9% vs. 27.3%, 95% CI 15.5%–43.0%; relative risk 0.224, 95% CI 0.068–0.744; *p* = 0.015) [11]. The Intelligent Detection Endoscopic Assistant (IDEA) system evaluates blind spots and image quality and generates an AI score that reflects the overall completeness of EGD. In a multicenter study involving 17,787 patients, the AI score was positively correlated with the detection rates of gastric cancer and EGC (*r* = 0.775, *p* = 0.003 and *r* = 0.756, *p* = 0.004, respectively) [12]. An AI‐system which combines EGD quality control with detection of early neoplasms (intelligent quality‐control system [IQCS]) was evaluated in a multicenter trial including 1840 patients, in which the GEN detection rate was significantly higher (6.1% vs. 2.3%, *p* = 0.0001), and had fewer blind spots (2.3 vs. 6.2, *p* < 0.0001) in the IQCS‐assisted group [13]. In terms of CAD‐EYE, AI for detection of esophageal squamous cell carcinoma and GEN, it is reported that the sensitivity, specificity, and specificity in the detection flag of GEN detection by using video clips were 95.5%, 86.1%, and 85.4% in the white light imaging (WLI) group, and 93.9%, 94.4%, and 93.9% in the linked color imaging (LCI) group, respectively. The sensitivities for EGC were 93.8% and 92.4% in the WLI and LCI groups, respectively [14]. To date, few trials have assessed AI‐assisted endoscopy in routine clinical practice. Available evidence indicates that AI can improve EGD quality by reducing blind spots and enhancing detection of GEN as a “second observer.” However, recent studies have also quantified measurable false‐positive rates. Additional prospective multicenter studies across diverse regions are needed to confirm the generalizability and clinical impact of AI in reducing GEN miss rates while managing false positives.

However, health economic evaluations of AI‐assisted EGD remain limited. In terms of short‐term cost implications, inspection times were slightly longer in the WISENSE group compared with controls (5.0 vs. 4.2 min, *p* < 0.001) [9] and in the ENDOANGEL group (5.4 vs. 4.4 min, *p* < 0.001) [10]. In an RCT using ENDOANGEL‐LD, the AI‐first group demonstrated a significantly lower biopsy rate and a higher positive predictive value during the first examination, while inspection time was unchanged between groups [11]. Similarly, in a multicenter study using the IQCS system, neither inspection time nor biopsy rate increased compared with controls across hospital types and endoscopist experience levels [13]. Overall, these findings suggest that AI‐assisted EGD may prolong inspection time by less than 1 min and does not increase biopsy volume, indicating that any short‐term cost impact is likely negligible. Regarding long‐term cost‐effectiveness, a Markov model analysis reported that AI‐assisted EGD may improve the cost‐effectiveness of gastric cancer screening with combined EGD and screening colonoscopy [15]. The cost‐effectiveness of AI‐assisted EGD is likely to vary depending on the accuracy, performance characteristics, and operational costs of individual AI systems. Ultimately, whether AI implementation can reduce gastric cancer mortality by improving early GEN detection remains uncertain and should be addressed in future prospective studies.

### 3.2 Computer‐Aided Diagnosis

Statement 2.1 We recommend developing CADx for differentiating among nonneoplastic lesions and GEN including gastric cancers to reduce unnecessary biopsies and endoscopic resections and improve the cost‐effectiveness. (Strongly agree 50.0%, Agree 50.0%).

Statement 2.2 CADx for GEN is likely to improve diagnostic accuracy of differential diagnosis between nonneoplastic lesions and GEN, but it is still challenging for CADx to predict tumor characteristics such as size, histological type, depth, and ulcerative findings for appropriate treatment decision making. (Strongly agree 33.3%, Agree 61.1%, Neither agree nor disagree 5.6%).

Statement 2.3 Health‐care delivery systems and authorities should evaluate the cost‐effectiveness of CADx for GEN to support its use in clinical practice. (Strongly agree 55.6%, Agree 38.9%, Neither agree nor disagree 5.6%).

Magnifying endoscopy with narrow band imaging (ME‐NBI) provides detailed evaluation of microsurface and microvascular pattern, allowing for differential diagnosis between noncancerous and cancerous lesions in the stomach [16]. Evidence indicates that CADx applied to ME‐NBI improves the differential diagnosis between nonneoplastic lesions and GEN, with preclinical still‐image and video studies demonstrating high sensitivity and specificity [17, 18, 19, 20]. In practice, however, distinguishing EGC from low‐grade gastric adenoma, that is typically classified as Category 3 in the Vienna classification, remains challenging [21]. Histological diagnosis using endoscopic forceps biopsy remains fundamental for the management of GEN. However, biopsy samples represent only a small portion of the lesion, and thus may fail to capture the full histological heterogeneity of GEN. This limitation highlights a potential advantage of ME‐NBI, which enables comprehensive evaluation of microsurface and microvascular patterns and may improve differentiation between gastric adenoma and EGC [22, 23, 24, 25]. Consistent with this, approximately 4%–30% of lesions initially diagnosed as adenomas on forceps biopsy are subsequently upgraded to gastric cancer following endoscopic resection [26, 27]. Current AI‐assisted endoscopic diagnostic systems have primarily focused on distinguishing gastric cancer from noncancerous lesions, including low‐grade adenomas. To date, no CADx system has been developed specifically to differentiate low‐grade gastric adenoma from EG. Furthermore, because comprehensive health economic evaluations remain limited, it is not yet possible to conclude whether CADx will effectively reduce unnecessary biopsies or endoscopic resections, or whether it will ultimately contribute to improved health‐care cost efficiency.

The guidelines state that endoscopic resection for EGC should be carried out when the likelihood of lymph node metastasis is extremely low, and when lesion size and site are amenable to en bloc resection [28]. The clinical indications for endoscopic resection are defined based on preoperative endoscopic and histopathological diagnosis of lesion size, depth of invasion, and presence or absence of ulceration [28, 29, 30]. Preoperative assessment of invasion depth is essential for selecting endoscopic versus surgical resection in EGC. Although some studies report high CADx accuracy for depth prediction, these findings are based on retrospective still‐image analyses and have not been validated in real clinical settings [31, 32]. Further prospective evaluation is needed. Moreover, no AI system currently supports tumor diameter measurement or ulceration assessment, both key factors in treatment decision‐making. Thus, continued innovation in AI is expected to enhance its ability to provide comprehensive therapeutic guidance in EGC.

The Japanese gastric cancer diagnostic guidelines recommend determining histologic type through a comprehensive assessment that combines biopsy findings with endoscopic evaluation [33]. Peripheral biopsies are advised when lesion margins are unclear, especially in undifferentiated‐type cancers with nonneoplastic epithelium. Staging ESD may also be used to avoid overdiagnosis, and endoscopic resection can be selected as a relative indication in EGC patients with some risk of lymph node metastasis, depending on prognosis, comorbidities, and posttreatment quality of life [28]. Because the costs of endoscopic resection and health‐care systems differ widely across regions, it remains uncertain whether CADx will consistently reduce medical expenses under such diverse conditions.

The role of CADx depends on the clinical purpose of EGD, which varies among countries. In Asian regions with a high incidence of gastric cancer, targeted biopsy is the standard approach for distinguishing cancerous from noncancerous lesions. In contrast, Europe and the United States primarily perform mapping biopsies to assess gastric cancer risk using OLGA and OLGIM classifications based on the updated Sydney system [34, 35]. Consequently, in Western practice, CADx is unlikely to replace biopsies unless its diagnostic performance clearly exceeds that of pathology. Surveillance intervals should be tailored to both population‐level gastric cancer incidence and individual risk. Although difficult to evaluate comprehensively, annual or biennial screening gastroscopy is desirable for detecting EGC in high‐risk groups. In several Asian countries, guidelines recommend screening endoscopy with targeted biopsy when abnormalities are found [36]. A recent Markov model analysis also supported the cost‐effectiveness of screening gastroscopy in these settings [37]. However, these findings may not apply to Western countries, where disease incidence, clinical practice, and health‐care economics differ substantially. Therefore, the cost‐effectiveness of CADx should be assessed with consideration of regional variation in endoscopy costs, the purpose of forceps biopsies, histological workflows, gastric cancer incidence, and health‐care systems.

### 3.3 Promotion of Research

Statement 3.1 We recommend conducting a broad range of high‐quality cost‐effectiveness studies to determine whether the implementation of AI provides meaningful benefits to populations and societies across different health‐care systems. (Strongly agree 66.7%, Agree 33.3%).

Statement 3.2 We recommend developing AI systems not only for GEN but also for all types of neoplastic lesions and other alert lesions throughout the upper GI tract. (Strongly agree 55.6%, Agree 44.4%).

Statement 3.3 We recommend developing AI for not only CADe and CADx of GEN but also risk stratification using background mucosa obtained by endoscopic images. (Strongly agree 55.6%, Agree 38.9%, Neither agree nor disagree 5.6%).

Statement 3.4 We recommend developing AI‐based risk stratification models that incorporate not only endoscopic images but also clinical, genomic, and epigenomic data to determine the optimal timing for EGD surveillance. (Strongly agree 55.6%, Agree 33.3%, Neither agree nor disagree 5.6%, Disagree 5.6%).

Despite being the fifth most common cancers, the prevalence of gastric cancer varies significantly in different regions globally [38]. As a result, universal recommendations on screening of gastric cancers would not be practical and should be tailored to the individual target populations. While national screening program was implemented in Japan and South Korea with evidence of reduction in gastric cancer related mortality [39], the latest multi‐society European guidelines (MAPS III) recommended screening program to be considered only if age standardized incidence rate is > 20 per 100,000 person‐years, although no European countries satisfy the threshold [40].

It is well known that EGC is frequently missed during endoscopy [5]. In lower incidence regions, training in detection of EGC could be even more challenging due to the lack of expertise. A recent single center tandem RCT in China demonstrated a reduction of gastric neoplasm miss rate from 27.3% to 6.1% when a CADe system (Endoangel‐LD) was used [11]. However, CADe may work differently with a lowered sensitivity in low‐prevalence situation with a psychological effect [41, 42]. So it is necessary to conduct clinical studies in low‐prevalence settings in order to show the usefulness of AI system. Recent meta‐analyses showed that AI systems had around 90% of sensitivity and specificity for diagnosis of EGC [43, 44]. While these studies showed encouraging results, most were based on retrospective still images. Differences in disease prevalence also substantially influence the probability of cancer in AI‐detected lesions, as many studies used high‐prevalence cohorts. Therefore, further high‐quality research is needed to evaluate the benefits of AI across diverse populations and health‐care systems.

A cost‐effectiveness analysis using a CADx system demonstrated an incremental cost‐effectiveness ratio (ICER) of USD$11,093 when applied to the Japanese population with moderate to severe *Helicobacter pylori* (Hp)‐associated atrophic gastritis [45]. Another study on the European population showed that performing gastroscopy with AI assistance combined with colonoscopy may be cost‐effective provided that the AI system achieved an accuracy of at least 93% and cost of ≤ 20€ [15]. Recently, a similar approach was also taken when considering the benefits of the AI system for colonoscopy polyp detection. Numerous RCTs have been published in the past decade, demonstrating superiority in adenoma detection rate when CADe systems were implemented. However, two recently published guidelines did not recommend routine use of such systems during colonoscopy, with one of them recommending against it [46, 47]. These recommendations stem from evidence that AI has not improved key outcomes such as colorectal cancer incidence or mortality while substantially increasing the burden of surveillance endoscopy. When considering AI implementation in upper GI endoscopy, it is likewise essential to focus on critical outcomes—particularly gastric cancer‐related mortality, cost, and resource implications.

A complete endoscopic examination in the upper GI tract should also include adequate inspection of multiple organs from the pharynx to duodenum. Esophageal squamous cell neoplasia (ESCN) and Barrett's esophagus related neoplasia (BERN) have already been the focus of research in the past decade. A recent meta‐analysis demonstrated a pooled sensitivity of 93% and 89% and specificity of 89% and 88% for ESCN and BERN, respectively [43]. Similar to AI studies on gastric neoplasia, the majority of the reported studies were conducted based on still‐image interpretation with a retrospective design. Two prospective single‐center clinical studies from Japan failed to demonstrate the superiority of a CADe system in detecting ESCN for a high‐risk population [48, 49]. On the other hand, a large‐scale tandem RCT in China demonstrated a reduction of the miss rate of ESCN from 6.7% to 1.2% [50]. In addition, numerous studies have also been reported on other non‐malignant upper GI pathologies, such as eosinophilic esophagitis [51], variceal detection and bleeding risk stratification [52], peptic ulcer classification [53], duodenal villous atrophy in celiac disease [54], and so on. Future research should also aim at the integration of AI systems designed for different purposes, so that clinicians could apply these AI systems based on the clinical indication and requirements.

Chronic Hp infection‐induced gastric atrophy (GA) and/or intestinal metaplasia (IM) is a high‐risk background mucosa especially for the development of intestinal‐type cancer. Background mucosa status was assessed according to the Kyoto gastritis classification, including normal mucosa, GA, IM, after Hp eradication, and so on [55]. Therefore, risk stratification using background mucosa obtained from endoscopic images, combined with AI, is important. For example, for endoscopic diagnosis of chronic atrophic gastritis (CAG) and OLGA staging assessment, Zhao et al. implemented a real‐time video monitoring model using U‐Net deep learning (DL) technology [56]. The diagnostic evaluation indices and the consistency between OLGA staging and pathological diagnosis of the model were superior to those of endoscopists, including the sensitivity (89.3% vs. 67.6%), specificity (90.5% vs. 70.2%), accuracy rate (89.9% vs. 68.9%), and area under the curve (AUC) (0.919 vs. 0.749, *p* < 0.001). This research confirmed that the DL model is capable of supporting endoscopists in the real‐time detection of CAG during gastroscopic procedures, while also enabling simultaneous recognition of patients with high‐risk OLGA stages (stages III and IV) [56]. In terms of an AI‐based system for the identification and stratification of GA, the AI system demonstrated a higher sensitivity than endoscopists (92.7% vs. 76.9%) at the image level, and it outperformed experts (94.9% vs. 85.9%) at the video level. In GA stratification, the system showed significantly better overall accuracy than experts (81.4% vs. 73.0%, *p* = 0.045). Its classification accuracy for non‐GA, mild, moderate, and severe GA was 80.0%, 77.4%, 83.3%, and 85.7%, respectively—comparable to expert performance and superior to that of senior and novice endoscopists [57]. Combining AI‐based endoscopic diagnosis with the Kimura–Takemoto classification enables more accurate assessment of GA severity and shows significant associations with Kyoto classification features such as irregular collecting venules, vascular patterns, and mucus in Hp‐positive gastritis. Atrophy severity differed significantly between high‐ and low‐risk groups in both pre‐ and post‐eradication states (*p* = 0.037 and *p* = 0.014). In the post‐eradication setting, the combined method achieved an AUC of 0.674, slightly higher than Kimura–Takemoto alone (AUC 0.661), indicating improved risk stratification [58]. Although AI combined with endoscopic images has demonstrated a higher sensitivity, specificity, and accuracy in risk stratification [56, 57, 58], clinical effectiveness remains limited by the molecular heterogeneity of gastric cancer. Integrative analyses combining clinical data, epigenetics, and genomic profiles can help overcome the limitations of endoscopic image–based assessment, enabling more precise risk stratification. For example, chronic Hp‐related inflammation can induce epigenetic alterations—particularly abnormal DNA methylation—even in endoscopically normal mucosa, although these methylation levels generally decline after Hp eradication [59]. In AI‐based endoscopy combined with molecular markers (including DNA methylation, mutations, and microsatellite instability) in detecting GEN, board‐certified endoscopists achieved the highest diagnostic accuracy (AUC: 0.931), followed by AI combined with miR148a methylation (AUC: 0.825), while trainees had the lowest performance (AUC: 0.588) [60]. Although AI combined with miR148a methylation did not achieve higher accuracy compared with board‐certified endoscopists, this result still suggests that AI‐assisted methylation analysis can be a valuable adjunct to the diagnosis of GEN. Additionally, to enhance traditional MSI screening methods, several studies have explored the application of DL for predicting MSI status using hematoxylin and eosin (H&E)‐stained histological images with around 0.8 of AUC [61, 62, 63].

To optimize EGD surveillance timing and improve gastric cancer risk assessment, AI systems should incorporate multimodal data beyond endoscopic images. This integrative approach could enable dynamic, individualized risk prediction by combining endoscopic findings with molecular‐level information. However, cost‐effectiveness must be considered, as molecular, epigenetic, and genomic testing remain expensive. A voter who disagreed with Statement 3.4 noted that, although risk stratification using clinical, epigenomic, and genomic data is important, developing AI specifically to determine optimal EGD timing is a relatively low priority at this stage.

## Author Contributions

Drafting of the “Computer‐aided Detection” section was carried out by N.K., G.H.K., and G.A.C. The “Computer‐aided Diagnosis” section was drafted by S.A., F.E., M.P., and V.N.A. The “Promotion of Research” section was prepared by H.C.Y., X.L., K.S., and M.D.‐R. M.F. prepared the “Introduction” and “Procedures for the Preparation of the Position Statements” sections. After critical review by all authors, M.F. integrated all sections and prepared the final manuscript for submission after voting and consensus. All authors made substantial contributions to the conception or design of the work; the analysis or interpretation of the literature for statements and explanations; the drafting of the manuscript or critical revision for important intellectual content; and the final approval of the version to be published. All authors also agree to be accountable for all aspects of the work, ensuring that questions related to the accuracy or integrity of any part are appropriately investigated and resolved.

## Funding

This study was self‐funded with support of World Endoscopy Organization.

## Conflicts of Interest

Mitsuhiro Fujishiro receives honoraria for lectures from Olympus Corporation, FUJIFILM Corporation, and AI Medical Service. Naomi Kakushima receives honoraria for lectures from Olympus Corporation and is a deputy editor in chief of Digestive Endoscopy. Seiichiro Abe receives research grants from Olympus Corporation and FUJIFILM Corporation and honoraria for lectures from Olympus Corporation, FUJIFILM Corporation, Boston scientific Japan, Johnson and Johnson, Medtronic, Daiichi Sankyo Corporation, AMCO, Takeda Pharmaceutical Corporation, and Creo Medical. Hon Chi Yip receives research grants from Research Grant Council, Government of Hong Kong SAR and Innovation Technology Commission, Government of Hong Kong SAR, and consultant fee from Olympus Corporation and honoraria for lectures from Olympus Corporation, Creo Medical, and Medtronic. The remaining authors declares no conflicts of interest.

## Data Availability

Data sharing not applicable to this article as no datasets were generated or analysed during the current study.
